# Counter-Punishment, Communication, and Cooperation among Partners

**DOI:** 10.3389/fnbeh.2016.00053

**Published:** 2016-04-05

**Authors:** Giulia Andrighetto, Jordi Brandts, Rosaria Conte, Jordi Sabater-Mir, Hector Solaz, Áron Székely, Daniel Villatoro

**Affiliations:** ^1^Institute of Cognitive Science and Technology-CNRRome, Italy; ^2^San Domenico di Fiesole, European University InstituteFiesole, Italy; ^3^Institut d'Anàlisi Econòmica-CSIC, Barcelona Graduate School of EconomicsBarcelona, Spain; ^4^Institut d'Investigació en Intel.ligència Artificial-CSICBarcelona, Spain; ^5^Department of Economics, University of BirminghamUK

**Keywords:** cooperation, norms, accountability, punishment, experiments

## Abstract

We study how communication affects cooperation in an experimental public goods environment with punishment and counter-punishment opportunities. Participants interacted over 30 rounds in fixed groups with fixed identifiers that allowed them to trace other group members' behavior over time. The two dimensions of communication we study are asking for a specific contribution level and having to express oneself when choosing to counter-punish. We conduct four experimental treatments, all involving a contribution stage, a punishment stage, and a counter-punishment stage in each round. In the first treatment communication is not possible at any of the stages. The second treatment allows participants to ask for a contribution level at the punishment stage and in the third treatment participants are required to send a message if they decide to counter-punish. The fourth combines the two communication channels of the second and third treatments. We find that the three treatments involving communication at any of the two relevant stages lead to significantly higher contributions than the baseline treatment. We find no difference between the three treatments with communication. We also relate our results to previous results from treatments without counter-punishment opportunities and do not find that the presence of counter-punishment leads to lower cooperation level. The overall pattern of results shows that given fixed identifiers the key factor is the presence of communication. Whenever communication is possible contributions and earnings are higher than when it is not, regardless of counter-punishment opportunities.

## Introduction

In this paper we use experiments to study the effects on cooperation of two types of communication. We do this in the context of a public good game with material punishment and material counter-punishment similar to but distinct from the one introduced in the seminal paper by Nikiforakis (2008)^1^. In our experiments participants repeatedly play a game involving a contribution stage, a punishment stage and a counter-punishment stage. We allow for communication separately at the contribution stage and the punishment stage as well as at both stages. Our work in this paper is part of our broader interest in studying the inter-play between punishment, communication and cooperation.

The possibility of inflicting punishment has been found to play an important role in improving cooperation in experimental environments (Ostrom et al., 1992; Fehr and Gächter, 2002; Sigmund, 2007; Dreber et al., 2008). However, it has also been shown that the presence and use of punishment can have negative side effects of various kinds.

The first drawback of the presence of punishment is that it may lead to breakdowns of cooperation. Gneezy and Rustichini (2000) provide an interesting example of this. In their experiment, a day care center started charging a small fine to parents who arrived late to pick up their children. Surprisingly, this resulted in an increase in the number of parents arriving late. Fehr and Rockenbach (2003) report similar results. They present evidence from a trust game, showing that trustees reciprocate less when they face the threat of punishment. Appealing only to extrinsic motivations, as punishment does, may lead to crowding out of intrinsic motivations to cooperate (Deci and Ryan, 1985, see Bowles and Polanía-Reyes, 2012, for a survey), and to “moral disengagement” (Bandura, 1991).

Second, undesirable effects of punishment can be caused by a dysfunctional use of it, like when punishment is directed at high contributors to a public good, a phenomenon also known as antisocial punishment (Anderson and Putterman, 2006; Herrmann et al., 2008; Gachter and Herrmann, 2011).

Third, the actual use of punishment can also lead to the reduction of welfare for the group involved due to an excessive use of resources to inflict punishment. In many experiments, the incurred costs of punishment outweigh the gains from increased cooperation (Fehr and Gächter, 2002; Egas and Riedl, 2008; Herrmann et al., 2008).

Our focus in this paper is on the effects of communication in the presence of *counter-punishment* opportunities. Our study was inspired by the seminal paper by Nikiforakis (2008), but our focus and some of the crucial elements of our design are quite different from his. First, our focus is on how in the presence of counter-punishment opportunities communication can affect behavior, whereas Nikiforakis (2008) showed that, in the absence of communication, the addition of counter-punishment opportunities can undo the positive effect of punishment opportunities on cooperation. Second, we look at a case of partners with fixed identification numbers in which participants can trace each other's behavior perfectly over time, whereas Nikiforakis (2008) studies the case of partners but without fixed identifiers. This distinction is important and may be the origin of some of our results. More on this below.

Nikiforakis (2008) is at the origin of the literature on counter-punishment. He finds that the presence of counter-punishment opportunities changes behavior substantially. First, it leads to a reduced use of punishment as a disciplinary device. The data show that approximately one quarter of all punishments is retaliated. The threat of revenge weakens subjects' willingness to punish free riders. Second, cooperation levels are lower than without counter-punishment (but higher than without both punishment and counter-punishment). Third, earnings are lower than earnings in the case with only punishment (which are again lower than without both punishment and counter-punishment).

In a complementary study based on a similar design, Denant-Boemont et al. (2007) create different information conditions to explore the relative strength of the effects of counter punishment as revenge toward those who punished them and as punishment directed at low punishers in the punishment stage. They find that people use counter-punishment as revenge, but also to punish those who fail to punish low contributors. Overall, they find that, as in Nikiforakis (2008), the possibility of counter-punishment leads to lower levels of cooperation.

Balafoutas et al. (2014) provide evidence on the effects of third-party punishment and counter-punishment in a one-shot three-player game. In their game, player B can take money from player A in a way that increases earnings inequality. Player C can then punish player B. The study compares behavior with and without a third stage in which player B can now counter-punish player C. The results show that many B players are willing to counter-punish and that the threat of counter-punishment has a large negative effect on the use of punishment. However, the existence of counter-punishment does not have any effect on selfish (non-cooperative) behavior by player B. In this sense, it is not a positive force in this case either.

Nikiforakis and Engelmann (2011) study the effects of punishment in an experimental environment with 10 rounds in which the number of punishment stages, and hence the length of a round, is endogenously determined by participants. They analyze the case where identification numbers change from round to round and the case where they are kept constant across rounds. It turns out that feuds, in the sense of participants punishing each other heavily across rounds, are rare in these experiments, because people use strategies that avoid the breakout of feuds. When identification numbers are constant over rounds (so-called long feuds) people's willingness to engage in altruistic punishment is greatly reduced. Cooperation rates decline over time leading also to a decline in earnings relative to a treatment in which altruistic punishment cannot be retaliated.

Engelmann and Nikiforakis (2015) study the effects of punishment in experiments with 30 rounds where, as in Nikiforakis and Engelmann (2011), the number of stages of each round is endogenously determined. They impose few restrictions on who can punish whom or when. They conduct a total of three treatments. In a baseline treatment fixed groups play thirty-rounds of a public good game without punishment opportunities in which identification numbers change from round to round. Two other treatments allow for multiple stages in each round. The only difference between them is that in one of them identification numbers vary over rounds whereas in the other the numbers are constant punishment. They report that they find no evidence of punishment leading to higher earnings despite the long horizon^2^.

Cinyabuguma et al. (2006) show that approximately one quarter of all punishments are retaliated. In their experiments, the existence of a counter-punishment stage reduces punishment of high contributors, but gives rise to efficiency-reducing second-order “perverse” punishment. They find that the existence of punishment does not lead to an increase in efficiency.

To the best of our knowledge there is no previous study on the effects of communication in the presence of counter-punishment opportunities and the aim of this paper is to fill this gap. In our view, the detrimental effects of punishment observed in the laboratory may be largely explained by the fact that they are used without any kind of explanation or justification. In natural environments punishment is not merely material but part of a broader phenomenon. It is typically meted out with reprimand, blame, criticism, gossip, and derision, enforcing strategies that naturally express social disapproval and signal norms (Ellickson, 1991; Boehm, 1999; Ostrom, 2005; Wiessner, 2005; Boyd et al., 2010), where by social norms we refer to prescribed behaviors shared and enforced by a community.

By contrast, laboratory studies have usually modeled punishment only by allowing players to castigate each other by the imposition of material costs. We think that when represented in these terms punishment is severely limited in its function of teaching people what the social norms governing the situation are and inform individuals about how they should behave (Cialdini et al., 1990; Crawford and Ostrom, 1995; Bicchieri, 2006; Conte et al., 2013)^3^. Purely material punishment does not make it possible to transparently and effectively convey the necessary information from which subjects may learn what constitutes socially prescribed conduct. This opacity of social norms does not help individuals to identify the prescribed conduct and may cause an overuse of punishment and trigger revenge as a reaction to punishment acts not recognized as legitimate^4^.

Recent experimental research has studied different ways of increasing punishment legitimacy and tested its effect in promoting the desired social outcome (Boyd et al., 2010; Faillo et al., 2013; Villatoro et al., 2014). In particular, Andrighetto et al. (2013) study how a particular type of communication interacts with punishment. They use a game with two stages, a contribution stage and a punishment stage, in which subjects may combine punishment with the communication of norms, by means of a message stating how much others should contribute paired with a message about why the required amount should be contributed. They provide experimental evidence that when combined with punishment, such messages prevent the detrimental effects of punishment both on cooperation levels and earnings and significantly reduce the use of costly punishment. Andrighetto et al. (2013) conclude that to effectively discipline people and achieve high levels of cooperation, punishment alone is not sufficient and individuals need to be placed in a setting in which norms are effectively communicated and properly understood. When combined with a normative message, punishment is perceived as a way of enforcing norms and not as a personally motivated action.

In this paper we focus on studying the effects of communication on cooperation in the presence of both punishment and counter-punishment opportunities. We see this as a natural step to a fuller understanding of the effects of communication on cooperation since—as pointed out by Nikiforakis (2008)—natural environments will typically include the possibility of counter-punishing. The public good game we use in this study has three stages, a contribution stage, a punishment stage, and a counter-punishment stage, common to all treatments. In our experiments groups are fixed throughout the duration of the experiment and, as already mentioned above, participant identification numbers are also kept constant across round. This is certainly not the only possible design choice, but we agree with the statement in Engelmann and Nikiforakis (2015, p. 565) that it is the most “realistic” case, since it most closely matches conditions outside the laboratory, where people who interact over time can often trace who did what in the past.

We conducted four experimental treatments: (1) punishment and counter-punishment by itself, (2) punishment combined with communication at the punishment stage and counter-punishment (3) punishment by itself and justification message at the counter-punishment stage, and (4) punishment combined with communication at the punishment stage and justification message at the counter-punishment stage.

More specifically, whereas in Treatment 1 there are no communication opportunities, in Treatments 2 and 4, subjects have at the punishment stage the possibility of combining punishment with a message asking for a specific contribution level, paired with a message providing a motivation why the required amount should be contributed. The message was: “One should contribute X, because: (a) In this way we are all better off, (b) It is what one should do, (c) If not, it will have consequences for you,” where the value of X was completed by the subject. In these treatments, we expect that subjects perceive punishment as legitimate, i.e., as a way of enforcing norms. Here we follow the design used in Andrighetto et al. (2013)^5^.

In Treatments 3 and 4, players who decide to punish somebody at the counter-punishment stage have to send a free-form message to the person that is being punished. Here we are interested in the effects of accountability, that is, of the implicit or explicit expectation that one may be called to justify one's beliefs, feelings, and action to others (see Tetlock, 1992)^6^. Xiao and Tan (2014) use a truth-telling game to study whether the use of punishment—not counter-punishment—is more likely to be consistent with social norms when there is a justification requirement. They find that the presence of justification pressure leads to punishment being more likely to be perceived as signaling norm violation and also to more truth-telling. Anti-social punishment may be curbed by the fact that it is hard to justify such a use as legitimate. In our study the focus is broader. Participants are not specifically asked to justify themselves, but are simply called to express themselves. We study whether the need for having to express oneself can help neutralize the potentially negative effects of counter-punishment.

We hypothesize that the presence of communication opportunities will lead to higher contribution, lower punishment, lower counter-punishment as well as higher earnings compared to the case without communication opportunities. We find that the three treatments involving communication at any of the two relevant stages lead to significantly higher contributions to the public good and higher earnings than the baseline treatment. We find no difference between the three treatments with communication. Used separately the two types of communication work equally well and we find no additional effect from using them jointly. Our results also show that there are no significant differences in the use of punishment, whereas there is some indication that counter-punishment is lower in the three treatments with communication.

The results are broadly consistent with the notions that receiving messages about what constitutes socially prescribed conduct helps individuals to coordinate on the desired outcome and influences punishment legitimacy and that the pure pressure of having to express oneself at the counter-punishment stage may enhance the norm salience by encouraging one to think about audience's beliefs and expectations about what one should do (Tetlock, 1985). However, our results also show that the uses of communication at the two different points in the experiment can't be neatly separated from each other. Communication at the final stage is often used to reinforce the communication of norms about contributions and not only in relation to the use of counter-punishment.

## Experimental design

### Procedures

Our experiments were conducted at the LINEEX laboratory of the University of Valencia. A total of 192 participants were recruited from a pool of undergraduate students from the University of Valencia and voluntarily participated in our experiment. Special care was exerted to recruit students from many different disciplines to increase the likelihood that the subjects had never met before. Each participant was allowed to take part in only one session. On arrival, participants were immediately led to separate cubicles. Instructions on general behavior in the lab and specific instruction about the game to be played were read by a mother tongue laboratory assistant. The experiment was programmed by using the z-Tree platform (Fischbacher, 2007)^7^.

### Treatments

In each of the four treatments, 12 fixed groups of four subjects interact over 30 rounds, divided in three blocks of 10 rounds. Players' identification numbers are fixed over the 30 rounds. As a consequence subjects can trace one another's behavior throughout the rounds^8^. We classify all actions in the punishment stage as “punishments” and all actions taken in counter-punishment stage as “counter-punishment.” Players' identification numbers are fixed over the 30 rounds. As a consequence subjects can trace one another's behavior throughout the rounds. We classify all actions in the punishment stage as “punishments” and all actions taken in the counter-punishment stage as “counter-punishment.” We acknowledge that in our design the distinction is somewhat blurred. However, we decided to maintain the distinction between punishment and counter-punishment, since in our case we have a rigid structure where the counter-punishment stage of a round is immediately followed by the contribution stage of a new round that can serve to “reset” the interaction.

The first 10 (1–10) and the last 10 (21–30) rounds are identical across all treatments, whereas rounds 11–20 are all distinct across treatments. Participants were told from the start that there were three blocks of 10 rounds, but were only informed about the rules for each block at the beginning of that block.

In every round of rounds 1–10 and 21–30, each member *i* of a group independently chooses an integer contribution level, C_i_, between 0 and 20, with the following payoff^9^:

$$\begin{array}{l} P_{i}=20-C_{i}+0.4\left(C_{1}+C_{2}+C_{3}+C_{4}\right) \end{array}$$

After each round all the members of the group are informed about the contribution levels of each of the other three members. In all four treatments behavior in rounds 1–10 is supposed to replicate with our procedures the well-known result that in a simple public goods game contribution levels invariably decay over time (Fehr and Gächter, 2000). Behavior in rounds 21–30 will inform us about possible spillover effects of the treatment conditions in rounds 11–20.

Rounds 11–20 of Treatment 1 consist of three stages. In stage 1 participants play the public goods game introduced above and then get feedback about the individual decisions of others. In stage 2 of each round participants can assign an integer amount between 0 and 10 punishment units to each of the other group members. Each assigned punishment unit costs the punished member 3 units and the punishing member 1 unit. After the punishment stage has finished participants get feedback about the punishment that has taken place. Each punished group member is informed about the IDs of all group members that have punished him^10^. In stage 3, called the counter-punishment stage, participants can punish again^11^. At the end of each round, each punished group member is informed about the IDs of all (counter)-punishers^12^. We will refer to this treatment as “punishment and counter-punishment.”

Rounds 11–20 of Treatment 2 consist of three stages, of which stages 1 and 3 are identical to those of Treatment 1. In stage 2 of Treatment 2 participants have, after each round, the opportunity to both assign costly punishment points as in Treatment 1 and send a normative message that has no payoff-consequences for any of the players of the group (we refer to it as the communication of norms). The content of messages has two components: the required contribution level “*One should contribute X”* (indicating the demanded token amount between 0 and 20) and a message providing a justification for contributing, which could be one of the following three options: “*because (1) in this way we are all better off; (2) it is what one should do; and (3) if not, it will have consequences for you.”* These options capture three different reasons for contributing: 1. achievement of a joint benefit; 2. a sense of duty; 3. a purely individualistic motive^13^. We will refer to this treatment as “sanction and counter-punishment,” where the term sanction refers to the combination of peer norm communication and material punishment (as in Giardini et al., 2010; Andrighetto et al., 2013).

Treatment 3 has stages 1 and 2 identical to those of Treatment 1 and a stage 3 which is different. If a player decided to use counter-punishment in stage 3, she was asked to write a message to each of the persons she punished (we refer to it as communication aimed to explain one's action).

Specifically, a chat box opened on the computer screen and the player had to introduce some text. It was clearly specified that the message had no payoff-consequences for any of the players of the group. In addition, in the instructions we wrote that the message was completely open, but that it was not allowed to identify oneself or to send offensive messages^14^. We consciously chose this minimalistic way of asking for a reaction, since any more explicit request or even the use of the word “justification” we considered to be too leading^15^. We will refer to Treatment 3 as “punishment and counter-punishment with message.” Treatment 4 combines the two communication possibilities of Treatments 2 and 3. We will refer to Treatment 4 as “sanction and counter-punishment with message.” Table 1 summarizes the information about the four treatments just discussed.

**Table 1:** **Treatments with counter-punishment possibilities**.

**Treatment number**	**Name**	**Characteristics**
1	Punishment and counter-punishment	No communication
2	Sanction and counter-punishment	Communication at the punishment stage
3	Punishment and counter-punishment with message	Communication at the counter-punishment stage
4	Sanction and counter-punishment with message	Communication at both stages

## Hypotheses

Our hypotheses are motivated by previous evidence about the effects of communication and by the literature presented in the introduction. We highlight here that our design is a rather complex one, involving actions at three stages and communication of different kinds at some of the three stages. We therefore focus our hypotheses on the effects of communication on the bottom line of the interaction, contribution and earnings levels. We formulate two sets of hypotheses about them.

Hypothesis 1:

*a. Contribution levels are higher in Treatment 2 than in Treatment 1*.

*b. Contribution levels are higher in Treatment 3 than in Treatment 1*.

*c. Contribution levels are higher in Treatment 4 than in all other treatments*.

Hypothesis 1a is inspired by the evidence in Andrighetto et al. (2013). In the two-stage design used there the possibility of communicating at the contribution stage leads to higher contributions. We conjecture that the possibility of counter-punishing in the current design will not undo this result, since the use of counter-punishment will be restrained by the fact that the norm of contributing will be made salient through communication and substantially followed. There will be only scant need to punish and anyway when used punishment will be perceived as legitimate, because it is aimed at discouraging the violation of the norm of contributing, and this will discourage counter-punishment^16^.

Hypothesis 1b is based on the notion that having to express oneself at the moment of counter-punishing may lead participants to think about others' expectations about socially prescribed behavior and to act in a way that avoids negative evaluations and reactions by others. Observe that we do not have a hypothesis pertaining to the comparison of contribution in Treatments 2 and 3, since we do not have an *a priori* about the relative strength of the two types of communication. Finally, Hypothesis 1c is based on the intuitive notion that communication taking place through two channels will have a stronger impact than when it only operates through one channel.

Hypothesis 2 is a translation of Hypothesis 1 into earnings levels, where we posit that the effects of communication will be strong enough to lead to higher earnings through a sufficiently high increase in contribution levels and the limited use of punishment and counter-punishment, with the corresponding limitation in the destruction of resources.

Hypothesis 2:

*a. Earnings levels are higher in Treatment 2 than in Treatment 1*.

*b. Earnings levels are higher in Treatment 3 than in Treatment 1*.

*c. Earnings levels are higher in Treatment 4 than in all other treatments*.

Hypotheses 1 and 2 pertain to the overall outcomes of the interaction in the different treatments. We now discuss possible hypotheses about punishment and counter-punishment. We start with counter-punishment since studying the interaction between communication and counter-punishment is the initial motivation for our work. In Treatments 3 and 4, we expect that the need to express oneself will lower the use of counter-punishment with respect to Treatment 1. The conjecture for Treatment 2 can't be based on the impact of the need to say something. Instead, we think that the fact of eliciting the norm of contribution and making it salient due to the possibility of sending a normative message will lead to high contributions and an overall climate of cooperation that will make punishment and consequently counter-punishment appear as pointless and unjustified. Moreover, in those rare cases in which punishment will be used, it will be interpreted as aimed to reinforce a norm and not only an idiosyncratic, or personally motivated, action. The perceived legitimacy of punishment will then reduce the likelihood and intensity of counter-punishment.

*Hypothesis 3: Counter-punishment levels are higher in Treatment 1 than in the other three treatments*.

With respect to punishment levels the results presented in Andrighetto et al. (2013) directly suggest that there will be more punishment in Treatment 1 than in Treatment 2, since the two treatments differ only in the presence of communication opportunities at the contribution stage. Predictions for Treatments 3 and 4 are less straightforward. As mentioned above, given the rather complex design of our experiment, our hypotheses are mostly about the bottom line of the interaction. However, our strong *a priori* about the power of communication leads us to predict that punishment will be less necessary and, hence, used less whenever communication is possible.

*Hypothesis 4: Punishment levels are higher in Treatment 1 than in the other three treatments*.

## Results

We begin by presenting the results pertaining to the levels of contributions to the public good and then move to the results about earnings levels and to the effects of punishment and counter-punishment.

### Contributions

**Figure 1:**
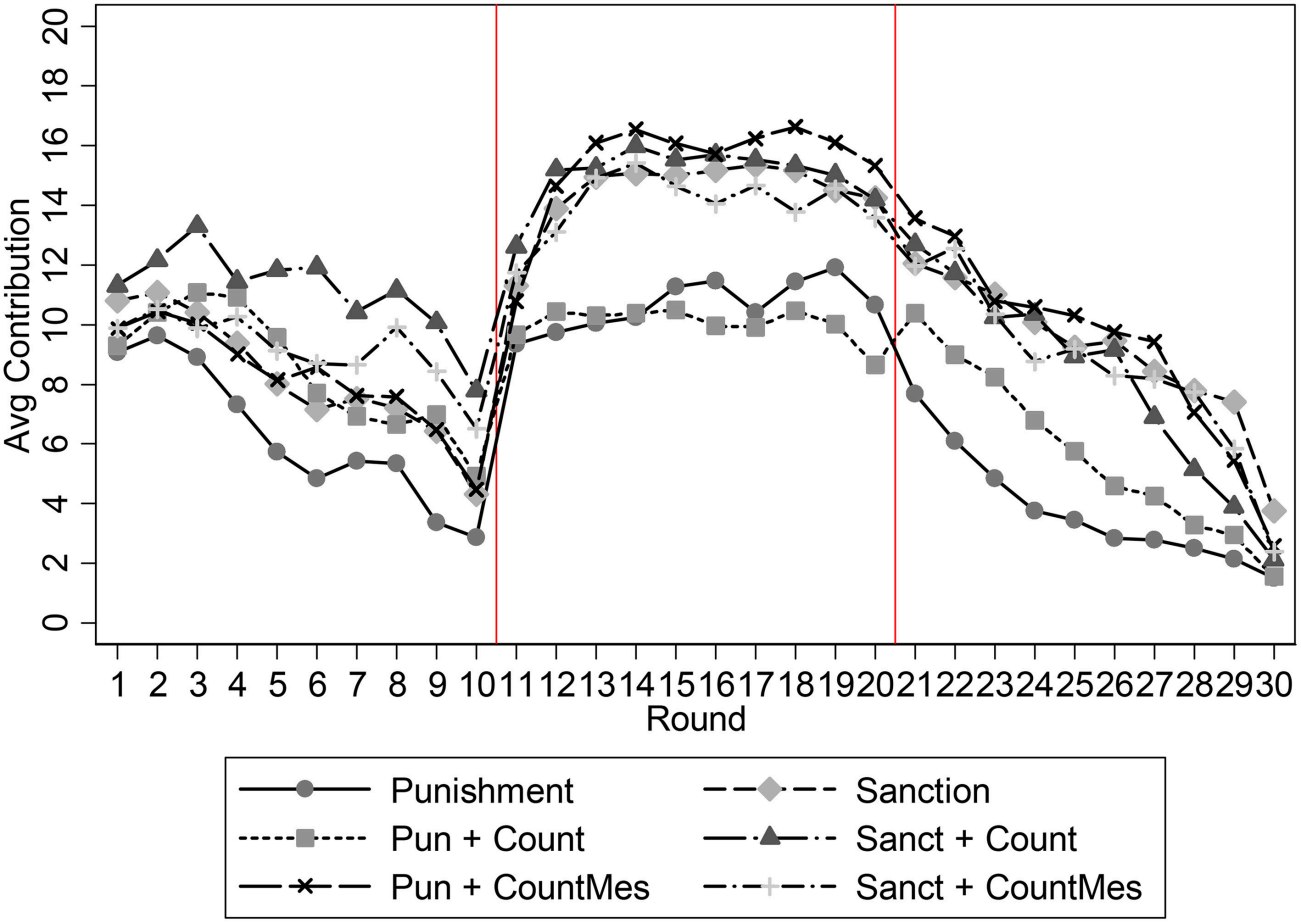
**Average contribution levels, comparing treatments with (Pun+Count; Sanc+Count; Pun+CountMes; Sanc+CountMes), and without a second punishment stage (Punishment and Sanction)**.

Figure 1 displays the contribution levels in a total of six treatments. For the moment focus on the four treatments involving counter-punishment. During rounds 1–10 contribution levels decline in all treatments, with average contributions being 8.44, 11.14, 8.22 and 9.19 in the *punishment and counter-punishment, the sanction and counter-punishment, the punishment and counter-punishment with message*, and *the sanction and counter-punishment with message* treatment respectively. Pairwise non-parametric tests show that contribution levels in Treatment 2 is higher than both in Treatments 1 and 3. Given that in rounds 1–10 all four treatments were identical any differences can only be random.

For rounds 11–20, one can see in the figure that three of the treatments have similar levels, while a fourth treatment has a noticeably lower level. Average contribution levels are 10.03, 15.03, 15.42, and 14.05 in the *punishment and counter-punishment, sanction and counter-punishment, punishment and counter-punishment with message required*, and *sanction and counter-punishment with message required* treatments respectively. In this case pair-wise non-parametric tests Mann-Whitney *U*-tests find that contributions in Treatment 1 are significantly lower than in each of the other treatments, whereas there are no differences between the other three treatments (*p*-values: 1–2 *p* = 0.0116; 1–3 *p* = 0.0056; 1–4 *p* = 0.0423; 2–3 *p* = 0.9020; 2–4 *p* = 0.7676; 3–4 *p* = 0.7119)^17^. In terms of the hypotheses presented in Results, we find support for hypotheses 1a and 1b, but not for 1c.

In the last 10 rounds, when punishment, normative message, counter-punishment and the opportunity to justify their own actions are switched off, contributions decay in all four cases to average levels of 5.68, 8.12, 9.24, and 8.53. Observe that contribution levels of the punishment and counter-punishment treatment are always the lowest. However, now only the difference between Treatments 1 and 3 remains significant.

Comparing average contribution levels between ten-round blocks within treatments we find that for Treatment 1 there is no significant difference between blocks 1 and 2 and while for all other treatments there is a significant increase from block 1 to block 2. We find that the three treatments involving communication at any of the two relevant stages lead to significantly higher contributions than the baseline treatment. The detrimental effect of allowing for counter-punishment is neutralized in the presence of communication possibilities. We find no difference between the three treatments with communication. Separately the communication of norms and being held accountable work equally well and we find no interaction effect from using them jointly.

Table 2 shows the results of hurdle model regressions. In both models the endogenous variable is the contribution level in block 2 (rounds 11–20). In Model 1 we only have categorical independent variables, where the case in which neither message is possible is the omitted category. First, one can see that the round variable is not significant for the contribution decision but is positive and significant at the 10% level for the contribution level. Second, the three variables corresponding to the treatments with messages all have positive and significant coefficients for the contribution level, but not for the contribution decision.

**Table 2:** **Contributions in block 2**.

**Independent variables**	**Model 1**		**Model 2**	
	**Contribution decision**	**Contributionlevel**	**Contribution decision**	**Contributionlevel**
Sanct+Count	0.320(0.315)	5.288(1.791)^**^	0.549(0.413)	5.906(1.854)^**^
Pun+CountMes	−0.054(0.326)	6.013(1.587)^***^	0.020(0.430)	6.783(1.659)^***^
Sanct+CountMes	−0.263(0.431)	4.863(1.898)^*^	−0.154(0.432)	5.707(2.023)^**^
Lagpunreceived			0.651(0.185)^***^	−0.317(0.178)
Treat2^*^lagpunreceived			−0.919(0.246)^***^	0.072(0.306)
Treat3^*^lagpunreceived			2.732(0.354)^***^	0.111(0.350)
Treat4^*^lagpunreceived			−0.710(0.196)^***^	0.086(0.272)
Lagcpreceived			−0.106(0.064)	0.800(0.127)^***^
Treat2^*^lagcpunreceived			3.095(0.243)^***^	−1.219(0.389)^**^
Treat3^*^lagcpunreceived			3.492(0.285)^***^	−0.946(0.286)^***^
Treat4^*^lagcpunreceived			−0.055(0.137)	−1.765(0.496)^***^
Round	−0.054(0.028)	0.169(0.076)^*^	−0.128(0.033)^***^	−0.002(0.079)
Constant	2.145(0.221)^***^	9.136(1.297)^***^	2.590(0.291)^***^	9.715(1.300)^***^
Wald χ^2^	9.74^*^		655.60^***^	
*N*	1840		1656	

* *p < 0.05*;

** *p < 0.01*;

*** *p < 0.001*.

*Hurdle model consisting of probit and truncated regressions. Estimated with cluster robust S.E.s according to group*.

Model 2 includes lagged punishment and lagged counter-punishment as well as interaction effects with all three treatments^18^. With respect to the pure treatment effects the results of Model 2 are similar to those of Model 1, while the round variable is now significantly negative for the decision and not significant for the level.

Moving to the effects of the lagged variables and their interactions we can see that for lagged punishment there are no significant effects on contribution levels, but there are on the contribution decision. For the contribution decision the pattern of the interaction effects is interesting. Observe first that lagged punishment has a significantly positive coefficient showing that in the baseline treatment without communication opportunities punishment has the expected effect. Note that the interaction effect between the Pun+CountMes variable and lagged punishment received is also significantly positive. This is the treatment without message at the contribution stage, so that the effect of lagged punishment seems intuitive in this case, in contrast to the negative interaction effects for the other two treatments. Testing for the sum of the coefficients, lagpunreceived+treat3^*^lagpunreceived, we find that it is positive and significant at the 1% level. In contrast, observe that the interaction effects for the other two treatments are significant but negative. In this case the corresponding sums of coefficients are not significantly different from zero. Hence, lagged punishment has an effect on contributions in the two treatments with no possibility for communicating norms at the contribution stage. This is consistent with the notion that in the absence of communication punishment is the instrument that is used to make contributions go up, whereas in the other treatments this is accomplished by communication itself.

The pattern of counter-punishment effects is harder to explain. We find that in the baseline treatment it has a significant positive effect on the contribution level but not on the decision. Moving directly to the sums of coefficients which capture the total effects of the other treatments, the results show that the coefficients for lagcpunreceived+treat2^*^lagcpunreceived and lagcpunreceived+treat3^*^lagcpunreceived are both significantly positive for the contribution decision but not for the level, whereas lagcpunreceived+treat4^*^lagcpunreceived has a significantly negative effect on the level but not on the decision.

Our main contribution in this paper is the study of environments involving counter-punishment possibilities. However, it is also interesting to relate our results here to those of two other treatments previously reported in Andrighetto et al. (2013). The experiments reported in that paper were conducted with the same subject pool and in the same lab as the ones corresponding to the treatments of Table 1. In addition, the instructions were the same except for a few differences. The two treatments from Andrighetto et al. (2013) that we refer to are called *punishment* and *sanction*. Both these treatments only have the contribution stage and the punishment stage, that is, they lack the counter-punishment stage. The treatment called *punishment* has a punishment stage just like Treatment 1 above and the treatment called *sanction* has a punishment stage just like Treatments 2 and 4 above, that is, participants can both impose costly punishment with material consequences both for the punisher and the punished subject and request a particular contribution level together with sending a normative message that is cost free.

For comparison Figure 1 also displays the contribution levels in the two treatments previously reported in Andrighetto et al. (2013) (*punishment* and *sanction*). For rounds 11–20, average contribution levels are 10.03, 15.03, 15.42, 14.05, 10.65, and 14.46 in the *punishment and counter-punishment, sanction and counter-punishment, punishment and counter-punishment with message required, sanction and counter-punishment with message required, punishment*, and *sanction* treatment respectively. Note also that in the *punishment* treatment the increase in average cooperation between blocks 1 and 2 is not large, a feature that can be attributed to the presence of fixed identifiers across rounds.

There is no significant difference between the two treatments with no communication (MW: 1-Pun: *p* = 0.8625). In contrast contributions in the *sanction and counter-punishment, punishment and counter-punishment with message required, sanction and counter-punishment with message required*, and *sanction* treatments are significantly higher by 50, 53.8, 40.1, 44.3% respectively, than in the *punishment and counter-punishment* treatment in which no communication is allowed (MW: 2-Pun: 0.0178; 3-Pun: 0.0056; 4-Pun: 0.0648, 1-Sanc: 0.0326).

At the same time, there are not significant differences between the *sanction* treatment, in which norm communication is allowed but only one stage punishment is present, and the other 3 treatments, in which both communication (i.e., message about the prescribed conduct and/or message justifying counter punishment) and a second punishment stage are allowed. Contribution levels are not always lower in the presence of counter-punishment opportunities (MW: 2- Sanc: *p* = 0.8294; 3- Sanc: *p* = 0.6033; 4- Sanc: *p* = 0.9509).

We can now summarize our findings about contribution levels in the following two results:

Result 1: When communication of any form is possible (i.e., communication of norms or communication aimed to explain one's action), contributions levels are significantly higher than when it is not possible.
Result 2: The presence of counter-punishment opportunities does not necessarily imply a decrease in contribution levels.

### Earnings

We move directly to looking at the earnings levels, since they constitute the bottom line of what we are interested in. If communication in the presence of counter-punishment only led to higher contribution levels, but not to higher earnings levels, one could say that effectively it is not very useful^19^. After this we will look at how punishment, counter-punishment and the two types of communication are used.

Table 3 shows average earnings levels per block and per treatments, including both the four treatments involving counter-punishment and the two treatments without it, previously reported in Andrighetto et al. (2013), where the latter are shown in italics. Starting with the comparisons between the four treatments involving counter-punishment we find that in block 1 earnings levels of Treatment 2 are significantly higher than in Treatments 1 and 3. This difference is purely random, since in block 1 the instructions are identical for all treatments.

**Table 3:** **Average earnings**.

**Treatment**	**Block 1**	**Block 2**	**Block 3**
1. Punishment and counter-punishment	25.07	22.64	23.41
2. Sanction and counter-punishment	26.69	26.55	24.87
3. Punishment and counter-punishment with message	24.93	25.66	25.55
4. Sanction and counter-punishment with message	25.51	25.71	25.12
*Punishment*	*23.75*	*21.43*	*22.25*
*Sanction*	*24.94*	*28.03*	*25.45*

We now move to block 2, which is our primary focus. Figure 1 reveals that in the initial rounds of block 2 contributions in the three treatments with communication are growing while in the punishment and counter-punishment treatment the average level of the block is reached more quickly. As will be shown below, punishment is also stronger in early rounds. We therefore test for treatment differences in earnings using the data from rounds 16 to 20. We find that there are no differences in earnings between the three treatments involving communication (i.e., *sanction and counter-punishment, punishment and counter-punishment with message, sanction and counter-punishment with message*). In contrast, in the *punishment and counter-punishment* treatment the average earnings are significantly lower than in the other 3 treatments, with *p* = 0.016, *p* = 0.004 and *p* = 0.074 for the comparisons with Treatments 2, 3 and 4 respectively^20^. In relation to the hypotheses presented in section, our evidence is consistent with hypotheses 2a and 2b, but not with 2c.

Comparing earnings levels of the four new treatments with those of the two treatments without counter-punishment, we find that earnings in the punishment treatment are significantly lower than in all treatments with communication and not significantly different than in the punishment and counter-punishment treatment. In contrast, earnings in the sanction treatment are significantly higher than in the treatments without communication opportunities at the punishment stage and the same as in the treatments with communication opportunities (MW: 1-Pun: *p* = 0.4529; 2-Pun: *p* = 0.0081; 3-Pun: *p* = 0.0179; 4-Pun: 0.0489; 1-Sanc: 0.0047; 2- Sanc: 0.2679; 3- Sanc: 0.0647; 4- Sanc: 0.2423)^21^.

Our main results with respect to earnings can be summarized as follows:

Result 3: The presence of counter-punishment opportunities only leads to a decrease in earnings when communication of any type is not possible.
Result 4: The possibility of communicating leads to significantly higher earnings, regardless of whether counter-punishment is possible.

### Punishment behavior and messages sent in the second stage

**Figure 2:**
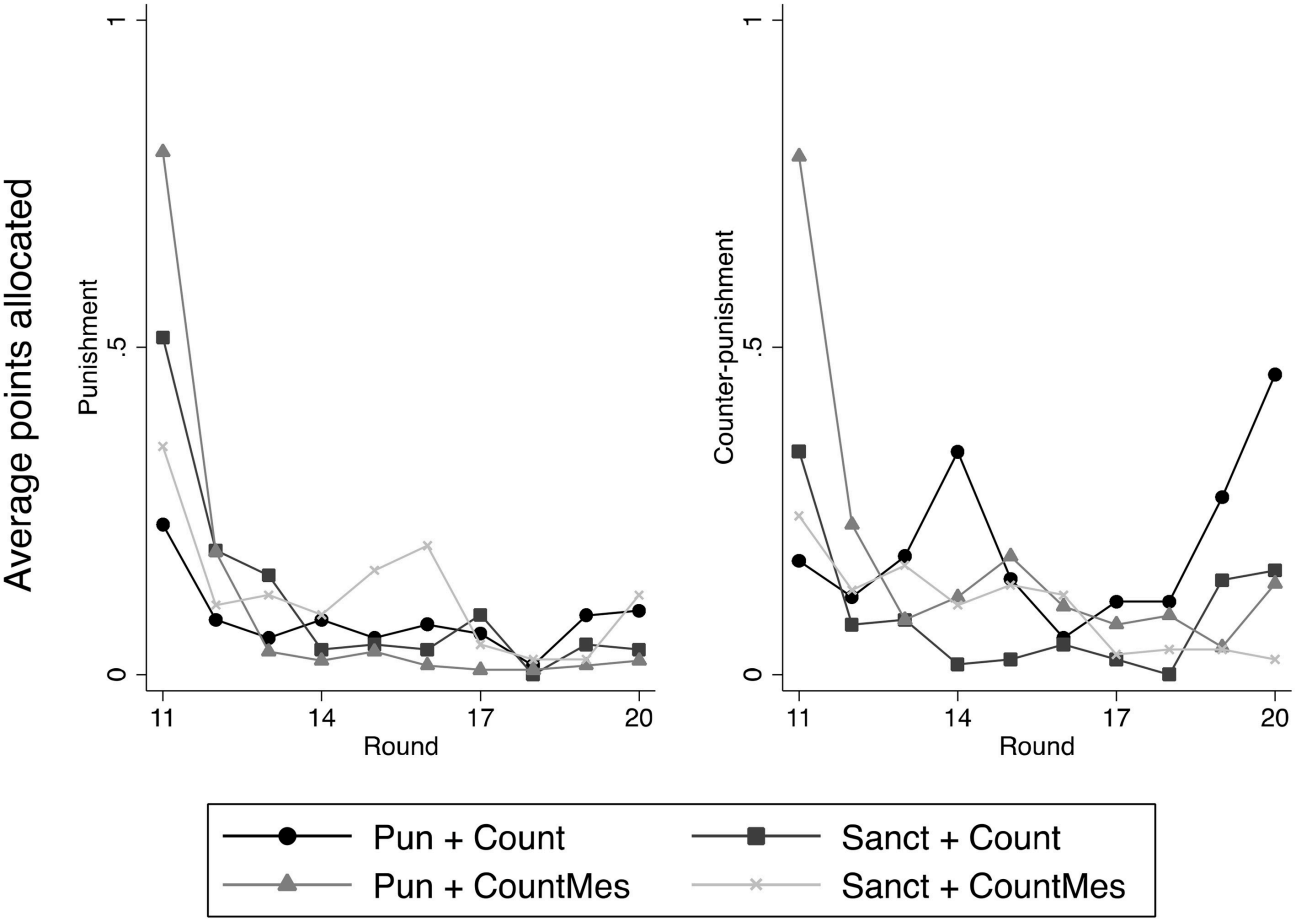
**Average number of punishment points and of counter-punishment points sent per decision in the four treatments with a second punishment stage, in rounds 11–20**.

The left panel of Figure 2 shows the average number of punishment points sent per decision for the four treatments with a counter-punishment stage. Overall, the number of points sent seems rather low, since the maximum for each decision is 10, with the level in round 11 being, in some treatments, somewhat higher than in the other treatments.

Table 4 shows the results of hurdle model regressions pertaining to punishment. The results for Model 1 show that there are no treatment effects for both the punishment decision and the punishment level, as suggested by the figure. In addition the variable “round” has a negative effect on the decision but not on the level, something which is not directly visible in the figure.

**Table 4:** **Punishment in block 2**.

**Independent variables**	**Model 1**		**Model 2**	
	**Punishment decision**	**Punishmentlevel**	**Punishment decision**	**Punishmentlevel**
Sanct+Count	0.092(0.151)	0.666(1.536)	0.068(0.146)	0.162(0.695)
Pun+CountMes	0.054(0.151)	0.344(1.850)	0.065(0.159)	0.475(0.943)
Sanct+CountMes	0.078(0.191)	1.945(3.065)	0.107(0.206)	1.365(1.115)
Own_Pos_Diff			0.057(0.017)^***^	−0.021(0.113)
Group_Pos_Diff			0.063(0.019)^***^	0.399(0.166)^*^
Round	−0.125(0.022)^***^	−0.378(0.360)	−0.119(0.022)^***^	−0.227(0.166)
Constant	−1.114(0.131)^***^	−5.898(8.922)	−1.469(0.122)^***^	−4.059(3.643)
Wald χ^2^	35.41^***^		199.55^***^	
*N*	5520		5520	

* *p < 0.05*;

*** *p < 0.001*.

*Hurdle model consisting of probit and truncated regressions. Estimated with cluster robust S.E.s according to group*.

In Model 2 we have added two regressors that need an explanation: “*Own_Pos_Diff”* and “*Group_Pos_Diff*.” Let *i* indicate the person who is the potential punisher and *j* a potential target. Then we define the variable of *Own_Pos_Diff* of person *i* in period *t* as *Own_Pos_Diff*_*i, t*_ ≡ *max*{0, *c*_*i, t*_ − *c*_*j, t*_} and $Group\_Pos\_Diff_{i,t}\equiv max\{0,(\sum_{h\neq j}c_{h,t})/(n-1)-c_{j,t}\}$ where *c*_*i, t*_ is the contribution of individual *i* in period *t* and *h* runs from 1 to 4 and refers to the group members. Thus, the variables represent the difference in contribution between the punisher and the potential target and the difference between the average contribution of the group (excluding the potential target) and the potential target's contribution, and we expect that both higher *Own_Pos_Diff* and *Group_Pos_Diff* are linked with more punishment. We find that both variables have a significantly positive effect on the punishment decision but not the level and *Group_Pos_Diff* has a significant positive effect on the punishment level. In addition, observe that in Model 2 there are no treatment differences in punishment decision and level.

We summarize:

Result 5: There are no differences in the decision to punish and in the punishment level between the four treatments.

#### Who punishes whom in the first and second punishment stages

**Figure 3:**
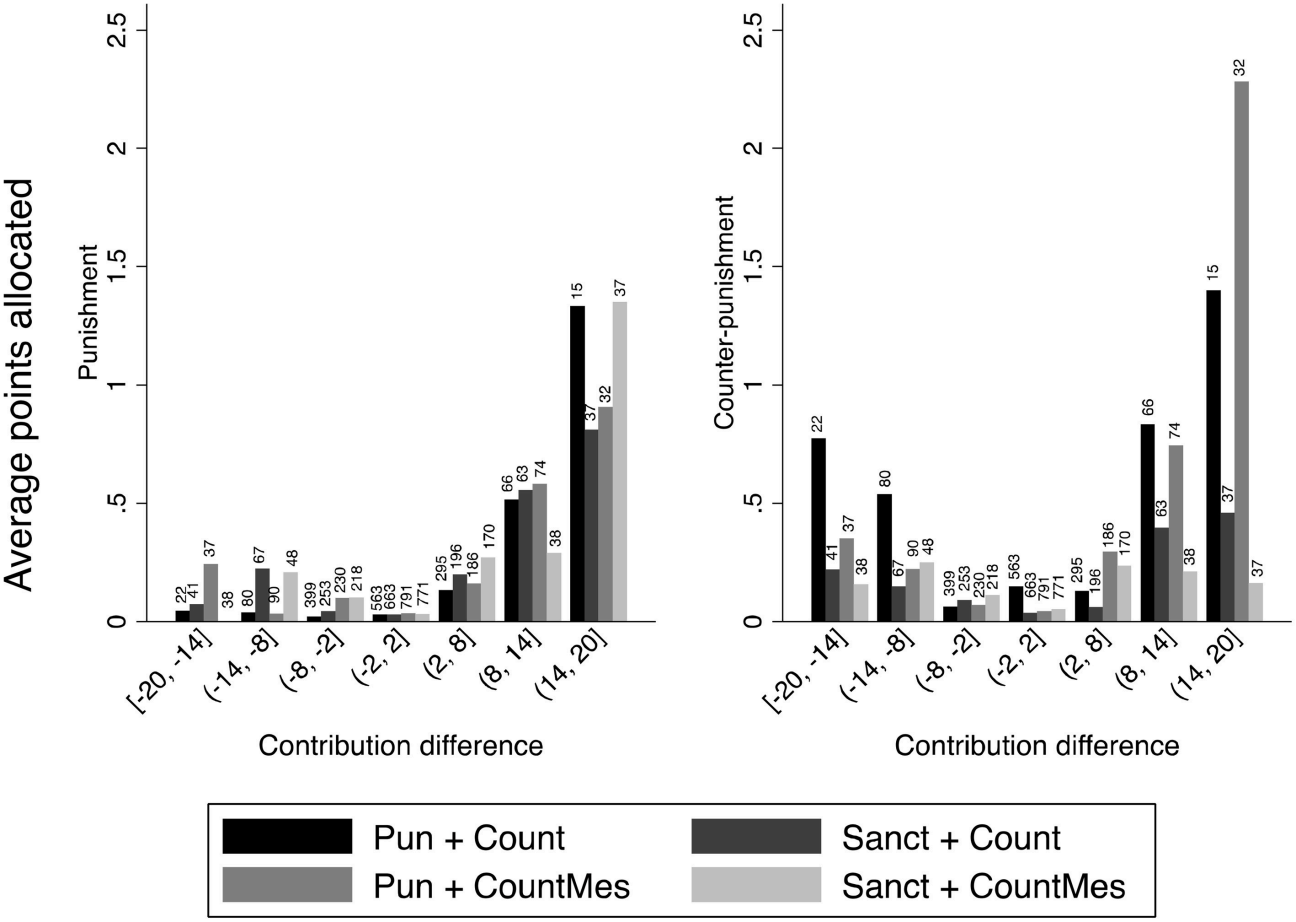
**Average number of punishment and counter-punishment points sent as a function of the differences between the contributions of the punisher and the potential target in the four treatments with a second punishment stage, in rounds 11–20**. The numbers above the bars indicate the total number of decisions pertaining to each case.

At this point we know that there are no significant differences in punishment levels between the different treatments, but it remains to be seen what the patterns of punishment are and whether there are any treatment differences. The left panel of Figure 3 shows how the number of punishment points assigned depends on the difference between the contributions of the punisher and of the punished. Observe first that anti-social punishment, i.e., punishment of participants who contributed relatively much, is rather infrequent. Second, observe that, for positive deviations, punishment is higher the more the target is behind. Finally, observe also that the overall patterns of punishment are not much different between the treatments.

#### Messages sent in the sanction and counter-punishment treatment

Recall that in the first punishment stage of the *sanction and counter-punishment* and in the *sanction and counter-punishment with message* treatment, subjects can send to the other group members both material punishment points and a message with the following content, choosing between options (1), (2), and (3): “One should contribute X (indicating the demanded token amount), because (1) in this way we are all better off; (2) it is what one should do, and (3) if not it will have consequences for you.” Table 5 displays the average required contribution level for the two relevant treatments. The average contribution levels required are 15.38 and 16.04, with no significant difference between the treatments. Interestingly, these are very close to the average contribution levels observed in these two treatments.

**Table 5:** **Average required contribution levels over rounds 11–20 in the sanction and counter-punishment and in the sanction and counter-punishment with message required treatments**.

**Round**	**11**	**12**	**13**	**14**	**15**	**16**	**17**	**18**	**19**	**20**
Sanct+Count	13.49	16.27	14.89	16.52	16.45	16.20	15.89	13.01	15.21	15.61
Sanct+CountMes	13.74	15.50	16.23	16.87	16.73	16.85	16.29	15.69	16.37	19.47

The frequencies of the three messages sent in stage 2 are very similar for the two treatments in which such messages are possible. Message 1 receives 71% and 72% in the two treatments respectively, whereas message 2 is sent between 10 and 20% of the times and message 3 is sent in 10% or less of the cases^22^.

Result 6: There are no significant differences in the use of messages in stage 2 between the two treatments in which such messages are possible: the sanction and counter-punishment treatment and the sanction and counter-punishment with message treatment.

### Counter-punishment behavior and messages sent in the third stage

The right panel of Figure 2 shows the average number of counter-punishment points sent per decision in the four relevant treatments. The data are in line with the ones for punishment shown in the left panel of Figure 2. Overall, counter-punishment is rather low.

Table 6 shows the results of hurdle models pertaining to counter-punishment. In Model 1 none of the treatment variables are significant (but observe that all the ones pertaining to the counter-punishment level have a negative sign). In Model 2, where we have added the deviation variables, all the treatment variables have a significant negative sign, indicating that counter-punishment in the Pun+Count treatment is higher than in the other three. Note also that in Model 2 some of the deviation variables are significantly positive.

**Table 6:** **Counter-punishment in block 2**.

**Independent variables**	**Model 1**		**Model 2**	
	**Counter-punishment decision**	**Counter-punishmentlevel**	**Counter-punishment decision**	**Counter-punishmentlevel**
Sanct+Count	−0.140(0.208)	−17.798(12.672)	−0.172(0.200)	−10.993(5.210)^*^
Pun+CountMes	0.303(0.210)	−23.398(14.865)	0.295(0.204)	−14.623(5.300)^**^
Sanct+CountMes	0.074(0.242)	−33.155(26.573)	0.093(0.245)	−18.148(8.594)^*^
Own_Pos_Diff			0.061(0.020)^**^	0.057(0.425)
Group_Pos_Diff			0.023(0.015)	0.854(0.431)^*^
Round	−0.082(0.019)^***^	1.051(1.253)	−0.074(0.018)^***^	0.671(0.542)
Constant	−1.220(0.193)^***^	−23.647(28.506)	−1.453(0.196)^***^	−13.957(10.228)
Wald χ^2^	28.26^***^		73.38^***^	
*N*	5520		5520	

* *p < 0.05*;

** *p < 0.01*;

*** *p < 0.001*.

*Hurdle model consisting of probit and truncated regressions. Estimated with cluster robust S.E.s according to group*.

In Table 7 we show results of hurdle models where to the independent variables of Table 6 we have added the two key variables “punishment received” and “lagged punishment received.” The former refers to the amount of punishment received from another member of the group at the punishment stage in the same period; the latter refers to the amount of punishment received from another member of the group at the punishment stage in the previous period. These variables are ego and target specific. Both variables are significantly and positively related with counter-punishment decision, but not with the level. Observe also that both in Models 1 and 2 the counter-punishment level in two of the treatments involving communication is lower than in the baseline.

Result 7: There is some indication that counter-punishment levels are higher in Pun+Count than in the other three treatments.

**Table 7:** **Counter-punishment taking into account punishment received**.

**Independent variables**	**Model 1**		**Model 2**	
	**Counter-punishment decision**	**Counter-punishmentlevel**	**Counter-punishment decision**	**Counter-punishmentlevel**
Sanct+Count	−0.255(0.209)	−16.520(7.802)^*^	−0.277(0.208)	−12.783(5.844)^*^
Pun+CountMes	0.117(0.214)	−17.624(7.984)^*^	0.111(0.204)	−14.171(6.711)^*^
Sanct+CountMes	−0.010(0.233)	−23.258(12.970)	0.011(0.241)	−17.518(9.331)
Punishment received	0.440(0.126)^***^	−3.349(3.070)	0.485(0.137)^***^	−1.158(2.034)
Lagged punishment received	0.142(0.031)^***^	−2.010(1.689)	0.162(0.027)^***^	−1.030(1.039)
Own_Pos_Diff			0.063(0.022)^**^	−0.361(0.377)
Group_Pos_Diff			0.032(0.017)	1.085(0.492)^*^
Round	−0.027(0.016)	1.091(0.950)	−0.019(0.017)	1.050(0.624)
Constant	−1.521(0.199)^***^	−13.341(12.370)	−1.797(0.214)^***^	−12.686(9.050)
Wald χ^2^	58.50^***^		82.05^***^	
*N*	4968		4968	

* *p < 0.05*;

** *p < 0.01*;

*** *p < 0.001*.

*Hurdle model consisting of probit and truncated regressions. Estimated with cluster robust S.E.s according to group*.

#### Who punishes whom in the counter-punishment stage

The right hand panel of Figure 3 shows counter-punishment as a function of the contribution of the punisher and of the punished (the target). One can see that there is a tendency to counter-punish more the higher the deviation of contributions, similarly to what is shown in the left hand panel for first stage punishment. Observe also that in this case there is also some indication that counter-punishment is higher for positive contribution deviations of the punished.

#### Counter-punishment and messages

**Figure 4:**
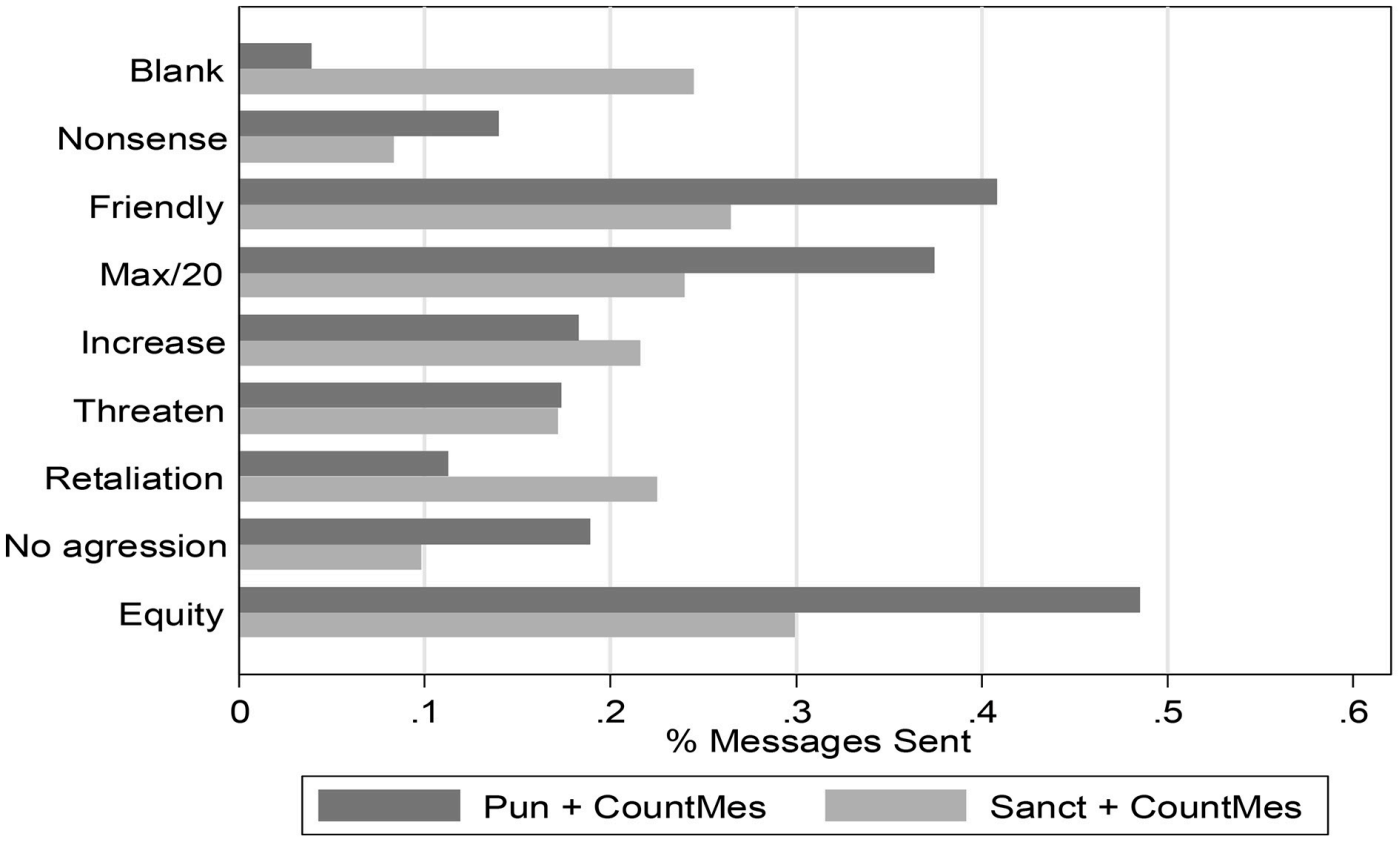
**Message content over rounds 11–20 in the punishment and counter-punishment with message required and in the sanction and counter-punishment with message required treatment**.

The frequency of use of messages at the counter-punishment stage is completely parallel to the use of counter-punishment, since a message has to be sent if and only if counter-punishment is used. Therefore, its use does not reveal any additional information about the use of counter-punishment to that already shown. Figure 4 shows the percentages of message sent, organized by content, in the sanction and counter-punishment and in the sanction and counter-punishment with message-required treatment, where every message has been classified into one of the categories^23^. There are no striking differences. However, one can see that the blank message is sent more frequently in Treatment 4. We conjecture that in this case an explicit message is perceived as less useful, since communication was already possible at stage 2, the punishment stage. Relatedly, observe also that the message asking for the maximum contribution of 20 tokens is more frequent in Treatment 3 than in Treatment 4, since communication in Treatment 4 involves precisely the specification of a required contribution level, which does not need to be repeated at this point. Note finally how friendly messages are quite frequent, something that has been observed in other experiments: when free-form messages can be used participants are typically friendly, perhaps to create an atmosphere conducive to cooperation^24^.

#### Punishment across stages

A final issue of interest is whether punishment is linked across stages. The fact that in our setting the punishment technology is the same in both stages and everybody is allowed to punish at both stages may suggest substitution between punishments across stages. Another question that arises is whether such links between punishments across stages depend on the treatment.

Table 8 shows the results of hurdle models for counter-punishment where with respect to Table 6 we have added the variable *Pun_Stage_2*, which corresponds to the number of punishment points each person allocated to each other group member in stage 2 of a given round. Since both the dependent variable and Pun_Stage_2 variable are target specific, Pun_Stage_2 indicates that people in a group punish and then counter-punish the same other group members in a given round. We have also added interaction terms between Pun_Stage_2 and each of the treatment variables; in Table 8 we use the abbreviation PS2 in the names of the interaction terms.

**Table 8:** **Counter-punishment taking into account the interaction between punishment and treatment variables**.

**Independent variables**	**Model 1**		**Model 2**	
	**Counter-punishment decision**	**Counter-punishmentlevel**	**Counter-punishment decision**	**Counter-punishmentlevel**
Pun_Stage_2	0.397(0.098)^***^	1.977(0.745)^**^	0.240(0.091)^**^	1.446(0.652)^*^
Sanct+Count	−0.160(0.208)	−9.191(5.148)	−0.176(0.203)	−8.150(4.533)
Pun+CountMes	0.178(0.205)	−9.854(5.148)	0.161(0.197)	−9.467(4.670)^*^
Sanct+CountMes	0.065(0.223)	−15.311(7.641)^*^	0.084(0.227)	−13.677(6.502)^*^
(Sanct+Count)^*^PS2	−0.050(0.149)	1.406(1.148)	−0.004(0.137)	0.758(0.872)
(Pun+CountMes)^*^PS2	0.632(0.191)^***^	0.252(0.875)	0.714(0.184)^***^	0.348(0.880)
(Sanct+CountMes)^*^PS2	−0.100(0.176)	2.784(1.695)	−0.037(0.181)	2.791(1.601)
Own_Pos_Diff			0.063(0.021)^**^	−0.054(0.217)
Group_Pos_Diff			0.002(0.015)	0.473(0.298)
Round	−0.053(0.015)^***^	0.810(0.494)	−0.051(0.015)^***^	0.752(0.394)
Constant	−1.397(0.185)^***^	−8.081(6.044)	−1.545(0.195)^***^	−7.993(5.205)
Wald χ^2^	114.35^***^		137.88^***^	
*N*	5520		5520	

* *p < 0.05*;

** *p < 0.01*;

*** *p < 0.001*.

*Hurdle model consisting of probit and truncated regressions. Estimated with cluster robust S.E.s according to group*.

Observe first that the impact of the three treatment variables is qualitatively the same as in the regressions shown in Table 6. In both Models 1 and 2 all treatment variables have a negative sign with some of them being significant. The results of Table 8 are in this sense consistent with our Result 7 where we write that there is some indication that counter-punishment levels are higher in Pun+Count than in the other three treatments.

With respect to the effect of Pun_Stage_2 we can see that in both models and for both the counter-punishment decision and the level the impact of this variable is significantly positive meaning that in our baseline treatment punishment and counter-punishment are complements and not substitutes. To examine the relationship between punishing and counter-punishing in the other treatments we examine the combination of main and interaction coefficients. Table 9 shows the results of statistical tests for the sums of the coefficients involving Pun_Stage_2. These show that for essentially all treatments, in both models and for both decision and level, punishing is likewise positively related to counter-punishing. The sole exception occurs in the decision to counter-punish in the Sanct+CountMes treatment in Model 2. These results potentially indicate that some participants have a general tendency to punish more than others.

**Table 9:** **Linear combination of coefficients from Model 1 and Model 2 in Table 8**.

**Coefficients**	**Model 1**		**Model 2**	
	**Counter-punishment decision**	**Counter-punishment level**	**Counter-punishment decision**	**Counter-punishment level**
Pun_Stage_2+ (Sanct+Count)^*^PS2	0.347^**^	3.383^*^	0.236^*^	2.204^**^
Pun_Stage_2+ (Pun+CountMes)^*^PS2	1.029^***^	2.229^**^	0.954^***^	1.794^**^
Pun_Stage_2+ (Sanct+CountMes)^*^PS2	0.298^*^	4.762^*^	0.203	4.237^**^

* *p < 0.05*;

** *p < 0.01*;

*** *p < 0.001*.

## Discussion and conclusion

We find that the presence of counter-punishment is only detrimental to contribution and efficiency when communication is not possible. The results of our study add to those of a number of other experimental results that show that the presence of communication very strongly affects behavior. A few examples of experimental work that highlight how communication matters in a variety of contexts are Charness and Dufwenberg (2006), Brandts and Cooper (2007), Ellman and Pezanis-Christou (2010), and Brandts et al. (in press).

Our results show that communication enhances the ability of individuals to self-govern and prevents the occurrence of undesirable effects. Our interpretation is as follows. The communication of norms both allows individuals to learn the prescribed conduct and to coordinate on it and punishment to be perceived as a way of enforcing norms and not a personally motivated action. It increases cooperation, punishment serves to maintain it, and the perceived legitimacy of punishment will reduce counter-punishment reactions. At the same time having to give account of the use of counter-punishment leads individuals to consider others' expectations about the prescribed conduct. Since counter-punishing those that legitimately punish constitutes a violation of a social norm, justification pressure allows eliciting this norm about punishment and makes individuals consider the consequences of its violation, thus limiting counter-punishment acts. Both types of communication help to render explicit the norms governing the experimental setting, norms regulating contribution and punishment, and prompt their compliance^25^.

However, our results also show that the uses of communication at the two different points in the experiment can't be as neatly separated from each other as laid out in the interpretation in the previous paragraph. Communication at the final stage is often used to reinforce the communication of norms about contributions and not only in relation to the use of counter-punishment. This is mainly due to the repeated nature of the interaction. Participants understand that what they say in the last stage of a round may influence others' behavior at the beginning of the next round. Indeed, of the messages that are sent at the last stage of the round many are not directly related to the use of counter-punishment, but refer to what the expected contribution level at the first stage of the game.

We find no difference in average behavior between the treatments involving communication: the communication of norms and accountability for the use of counter-punishment have the same overall effect and even the joint use of both communication possibilities does not improve on the separate use. We find no differences in punishment behavior and only some minor differences in counter-punishment behavior between all four treatments, attributable to the use of fixed identifiers across rounds. Moreover, if we consider the four treatments with counter-punishment and the two without it, first reported in Andrighetto et al. (2013), we can say that the factor that organizes contribution and efficiency levels is the not the presence of counter-punishment but the presence of communication that allows norms to be elicited and made salient and individuals to reason on them and consider the consequences of their violations.

## Ethics statement

Our experiment is about decision-making and involves no physical intervention. All our experimental sessions were conducted with the informed consent of all adult participants, who knew that they were free to withdraw from participation at any time. Individuals invited to one of our sessions had previously voluntarily registered in the LINEEX laboratory of the University of Valencia database. To do that they had to go to LINEEX website. On that website the rules of the lab were available. Informed consent was indicated by electronic acceptance of an invitation to attend an experimental session. The voluntary registration in the electronic database documents participants' acceptance. The experiments were conducted following the procedures established by LINEEX laboratory of the University of Valencia. Our study was approved by the Director of the LINEEX laboratory (Professor Enrique Fatás at the time) at an ethics review and project proposal meeting that is required for all experiments conducted at the LINEEX facilities.

## Author contributions

All authors listed, have made substantial, direct and intellectual contribution to the work, and approved it for publication.

### Conflict of interest statement

The authors declare that the research was conducted in the absence of any commercial or financial relationships that could be construed as a potential conflict of interest.

## Footnotes

^1^From now on the terms punishment and counter-punishment will always be meant in the material sense, i.e., as the imposition of material losses on others.

^2^Nikiforakis et al. (2012) also study the emergence of feuds but in an environment with normative conflict, which arise from the fact that individuals derive different benefits from the public good. They find that in such a setting punishment is much more likely to trigger counter-punishment and start a feud.

^3^For an example of the powerful effects of communication in another context see Brandts et al. (in press).

^4^As defined by Tyler (2006), legitimacy is “a psychological property of an authority, institution, or social arrangement that leads those connected to it to believe that it is appropriate, proper and just” (p. 375) and is a fundamental element governing social interaction. Following Zelditch (2001), we suggest that something is legitimate if it is in accord with the norms shared within a group and that a natural way of legitimizing the use of punishment is by identifying it as a way of reinforcing norms and righting in violators.

^5^Our procedure uses pre-formulated messages. This may lead to different results than full-fledged free-form communication.

^6^In other contexts it has been shown that this expectation affects people's decision-making (Lerner and Tetlock, 1999; Vieider, 2011; Brandts and Garofalo, 2012).

^7^The instructions for Treatment 4 can be found in the Appendix in Supplementary Material.

^8^A reviewer asks us to stress that our results are only for a partners protocol with fixed identifiers across rounds. We think that the abstract of the paper makes this now very clear from the start.

^9^This function is the same as the one used in Fehr and Gächter (2000) and Nikiforakis (2008).

^10^Participants could see the full vector of contributions but only the punishment, counter-punishment and messages they received. In the instructions it said: “At the end of each round (period) you will receive information about the contributions of each of the members of the group, the total contribution of the group, the punishment points you that each of the other members of the group have sent you (in each of the phases), the total points received in each phase, the messages each member of the group has sent you, the profit that you obtained in the round and the accumulated profit up to this point.” This is different from Nikiforakis (2008) where, after each round, each subject was only informed about the total quantity of punishment points assigned to him. Our procedure is the same as in the “Revenge” treatment in Denant-Boemont et al. (2007).

^11^The exact same punishment technology than in the punishment stage applies: participants can assign an integer amount between 0 and 10 punishment units to each of the other group members. Each assigned punishment unit costs the punished member 3 units and the punishing member 1 unit.

^12^There were no restrictions on who could punish who in both stages 2 and 3. This is again different from Nikiforakis (2008) in which each individual had the opportunity to punish only those who punished him.

^13^We consider the third message to appeal to an individualistic motive, since it refers to payoff losses.

^14^Indeed, it was possible for a player to type only one character into the chat box for the message to be acceptable, although we did not make this explicit to participants.

^15^Xiao and Tan (2014) use a somewhat more pushy way of asking for a reaction. Their formulation in the instructions was: “Person X must write a message to explain his/her and the explanation must be related to Person Y's behavior.” Our procedure is more in the sense of asking somebody “what do you have to say?”

^16^Assuming rationality, selfish preferences, and common knowledge of rationality the sub-game equilibrium prescribes no counter-punishment, no punishment and no contributions in the first stage. This holds for the stage game as well as for the finitely repeated game, which can be shown by backward induction. In addition, both types of communication have no effect.

^17^In all non-parametric tests reported in the paper the unit of observation is the group. Here group-level contributions are averaged across rounds within a block.

^18^For ease of notation in the table we have changed the names of the treatments and now use numbers. See Table 1.

^19^As already mentioned in the introduction, in some environments the presence of punishment opportunities of different kinds has led to higher contributions, but lower earnings.

^20^In block 3 earnings are still significantly higher in Treatments 3 and 4 than in Treatment 1, but earnings in Treatment 2 are not higher.

^21^Observe that the result that the mere addition of counter-punishment opportunities has not effect on earnings levels (MW 1-Pun = 0.4529) is not in line with the result in Nikiforakis (2008). The likely explanation is that we use fixed identifiers across rounds.

^22^The results for the messages are very similar to the ones reported in Andrighetto et al. (2013) for the sanction treatment.

^23^The messages were coded independently from the authors. The labeling of the different message categories is self-explanatory, except perhaps for Max/20 which refers to asking for the maximum possible contribution of 20.

^24^A reviewer asks about the relation between the threatening message at the second stage and the high proportion of “friendly” messages in the counter-punishment stage. Note that the threatening message is the least frequently used of all three and is sent in < 10% of cases. One possible connection between the messages is that participants may have a tendency to want to establish a friendly rapport between them. To do this it seems reasonable to avoid the threatening message at the second stage and to be friendly at the third stage.

^25^A reviewer of the paper considers that our design does not allow us to claim that communication enhances the ability of individuals to self-govern themselves, because the messages we use are too constrained. We understand that the use of pre-formulated messages may be a limited way to communicate norms, but we do think that using them is an acceptable way of studying the effects of norms. Future research could try to go into detail in analyzing the impact of different ways of communicating norms
