# Supplementary material for: Counter-Punishment, Communication, and Cooperation among Partners
Source: Front Behav Neurosci. 2016 Apr 5;10:53. doi: 10.3389/fnbeh.2016.00053 (PMC4821197; doi:10.3389/fnbeh.2016.00053)
Supplement: Supplementary file 1 [file DataSheet1.docx]

**APPENDIX**

**Instructions (for the Sanction and Counter-Punishment with Message treatment, Block 2)**

The purpose of this experiment is to study how individuals make decisions in certain contexts. The instructions are simple and if you follow them carefully you will be paid a cash amount of money privately, since nobody will know about the earnings of the other participants. You can ask questions at any moment by raising your hand. Apart from these questions, any type of communication between you is not allowed and may lead to exclusion from the experiment.

1. These are the instructions for the second block, which consists of 10 rounds.
2. In each and every round you are part of the same group of 4 participants in which you have been until now. Each of the members of the group maintains his ID (ID 1, ID 2, ID 3 OR ID 4).
3. In each round you have to make several decisions. The first decision is identical to those you have made until now: to decide how many of the 20 ECUs that you receive each round you assign to a Group Fund, with the rest being automatically assigned to a Private Fund. Your preliminary profit is calculated like in the first block of the experiment.
4. Once all contributions are known each member of the group has the possibility of sending punishment points (between 0 and 10) to each of the members of the group. Player 1 decides how many points to send (between 0 and 10) to 2, how many to send (between 0 and 10) to 3 and how many (between 0 and 10) to 4; Player 2 decides how many points to send (between 0 and 10) to 1, how many to send (between 0 and 10) to 3 and how many (between 0 and 10) to 4; etc.
5. Sending punishment points has two consequences:
6. For each point that you send to another member of your group you reduce by 3 ECUs his preliminary profit. If you send 0 points to another participants you do not modify his profit, but if you send him 1 point you reduce his profit by 3 ECUs, if you send him 2 points you reduce it by 6 ECUs etc. Observe that you have to write a number between o and 10 for each of the members of the group and that all the points received by each gplayer will be used to determine the reduction of his preliminary profit.
7. Sending points has a cost for whoever sends them. Each point sent reduces the preliminary profit of the sender by 1 ECU.

6. In addition, at the same time that he sends punishment points each group member has the possibility of indicating to each of the other participants what right behavior he considers right, by completing the following sentence and marking one of the following options:

*One should contribute X, because:*

1. *In this way we are all better off*
2. *It is what one should do*
3. *If not, it will have consequences for you.*

This message has no direct effect on your payoffs or the payoffs of the receiver of the message.

7. The third decision consists again in, after having been informed about the punishment points received from each of the other players, sending punishment points (with the same consequences as in the previous phase).

8. In addition in this phase, each member of the group has to send a message to each of the other participants to whom he send points (in case of sending 0 to any of the other participants it will not be possible to send them a message). This message has no direct effect on your profit or on the profit of the recipient.

Attention: The message is open, but you are not allowed to identify yourself (or to provide information which facilities identification) or to send messages that could be offensive. In both cases the participant will be excluded from the experiment.

9. Your profit in a round is the result of applying to your preliminary profit the reduction associated with the total amount of points that you have received (between 0 and 60 in both phases) and with the subtraction of the total cost of the points that you have sent in both phases. That is:

Final Profit = Preliminary Profit – Reduction due to total points received – cost of total amount of points sent.

10. Observe that you final profit can be negative if the cost of the points you send are higher than the preliminary profit reduced by the points received. These losses can in any case be compensated by profits in another round of this block.

11. At the end of each round you will receive information about the contributions of each of the members of the group, the total contribution of the group, the punishment points sent to you by each of the other members of the group (in each of the phases), the total of points received in each phase, the total of points sent in each phase, the messages sent to you by each of the other members of the group (in each of the phases), the profit you obtain in this round and the accumulate profit up to this point.

12. At the end of the experiment you will be paid in cash and privately your accumulated payoffs for the whole experiment at the exchange rate of 40ECUs = €1
